# How thoracic surgeon manage tracheal tumors in African country? (Case series)

**DOI:** 10.1016/j.amsu.2019.06.007

**Published:** 2019-06-18

**Authors:** Sani Rabiou, Badredine Alami, Boubacar Efared, Marwane Lakranbi, Hicham Harmouchi, Rim El-Amrani, Mounia Serraj, Abderrahim El-Bouazzaoui, Yassine Ouadnouni, Nabil Kanja, Mohamed Smahi

**Affiliations:** a Department of Thoracic Surgery, CHU Hassan II, Fez, Morocco; b Department of Radiology, CHU Hassan II, Fez, Morocco; c Department of Cytopathology, CHU Hassan II, Fez, Morocco; d Department of Pneumology, CHU Hassan II, Fez, Morocco; e Department of Anaesthesiology and Resuscitation A4, CHU Hassan II, Fez, Morocco; f Faculty of Medicine and Pharmacy, Université de Niamey, Niger; g Faculty of Medicine and Pharmacies, Université Sidi Mohamed Ben Abdellah, Fez, Morocco

**Keywords:** Tracheal tumors, Tracheal stenosis, Adenoid cystic carcinoma, Rigid bronchoscopy, Tracheal endoprosthesis, Surgery

## Abstract

**Introduction:**

Tracheal tumors are a rare pathological entity whose diagnosis is usually delayed by clinical latency. Surgery, which consists of a tracheal resection-anastomosis with or without reconstructive reconstruction, remains the treatment that ensures the best long-term survival.

**Methods:**

This is a retrospective study about 8 patients admitted in the department of thoracic surgery of Hassan II's university hospital of Fes for tracheal tumors management during 7 years time (December 2010 to December 2017).

**Results:**

There were 6 men and 2 women with an average age of 44.4 years ranged from 17 to 65 years, 4 were smokers. Dyspnea was the main trigger sign. Seven (7) have undergone bronchial fibroscopy diagnostic with a finding of budding process in 5 patients, the middle of the trachea is often concerned in 3 patients, obstructing the lumen of the trachea in almost all patients. The treatment in all patients was surgical with an intubation via the operative field, 4 trachea resection-anastomosis, 4 plasty (Lateral resection with V plasty, Kergin's plasty, Mattey's tracheobronchial anastomosis and widened V-resection to the carina). The most common histological type in our series was Adenoid Cystic Carcinoma in 2 of our patients. For the other patients we have found squamous cell carcinoma (1 case), adenocarcinoma (1 case), atypical carcinoid tumor (1 case), low grade mucoepidermoid carcinoma (1 case), an adenoma pleomorph (1 case) and endotracheal goiter (1 case). The operative follow-up was simple in 7 of our patients, all of whom underwent post-operative fibroscopy within an average of 9 days. Two cases of post-operative recurrent palsy had been observed, all of which had progressed well under treatment. We have noted 2 deaths, including one at day 4 post-operative, and the other died from complications of post-radiation tracheal stenosis. Back to 32 months' average follow-up, we have enregistered a case of a distant relapse by cervical lymph node metastasis in one patient, 5 years after surgery.

**Conclusion:**

Primary tumors of the trachea remain of reserved prognosis with 5-year survival of 57% of all histological types combined. Computed tomodensitometry and tracheobronchial fibroscopy remain the means of reference exploration in the diagnosis and assessment of surgical resectability.

## Highlights

• Primary tumours of the trachea are rare and are usually malignant in adults and benign in children.
• Benign tracheal tumours are usually misdiagnosed as asthma or chronic.
• Malignant tumours are often diagnosed earlier than benign tumours and patients thus often present with locally advanced disease.
• Various surgical techniques can cure primary tumours of the upper airway.

## 1 Introduction

Primary tumors of the trachea are rare, representing 2% of all tumors of the upper airways [1]. The majority of these tumors are malignant in adults whereas they are benign in 90% in children [2]. The most common malignant tumors are squamous cell carcinoma and cystic adenoid carcinoma, called cylindromes in the pass [3]. The diagnosis is often late, in patients who are often mistakenly treated for asthma or other obstructive respiratory diseases. The treatment which is above all surgical has seen notably evolved concerning the technique of tracheal mobilization both in the mastery of anesthesia [4]. Endoscopic treatment is indicated whenever surgery is contraindicated or pending. Through a 7-year retrospective study, we wanted to share our experience in the surgical management of this rarely reported pathological entity.

## 2 Materials and methods

This is a retrospective study about 8 patients admitted in the department of thoracic surgery of Hassan II's university hospital of Fes for tracheal tumors management during 7 years' time (December 2010 to December 2017). After analyzing all patients' files who underwent tracheal surgery during this period, we have retained only 8 files.

- been over 16 years' old
- the tracheal lesion must be of tumoral origin
- the procedure must include resection-anastomosis of the trachea
- the tumor must be confirmed by anatomo-pathological examination of the excision part
- follow-up of the patient must be performed by the thoracic surgeons who carried out the procedure.

We excluded patients operated for tracheal lesions in emergencies (including wounds and tracheal rupture) and those who underwent surgery for benign tracheal stenosis including post-intubation. We have also collected data for each patient on: age, sex, history, symptoms, clinical and radiological examination results, endoscopic outcome, anesthetic and surgical management, the nature of the histological act as well as the evolution of the patients in the immediate postoperative period and far away. The work has been reported in line with the PROCESS criteria [5].

## 3 Results

### 3.1 Clinical aspect

**Fig. 1:**
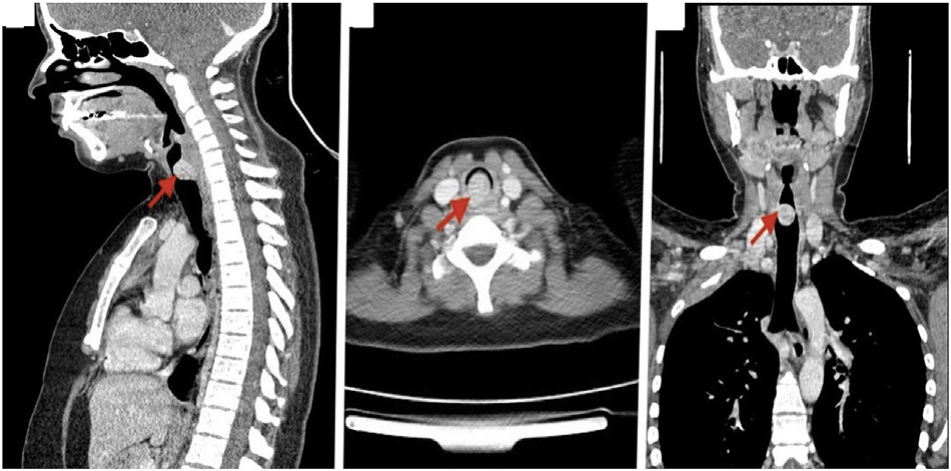
Thoracic CT scan with sections showing endotracheal tumor.

**Fig. 2:**
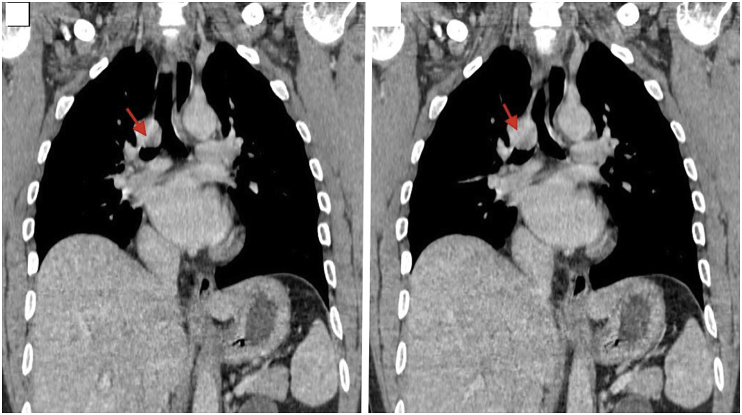
Thoracic CT scan frontal section showing tumor involving the tracheobronchial area.

**Fig. 3:**
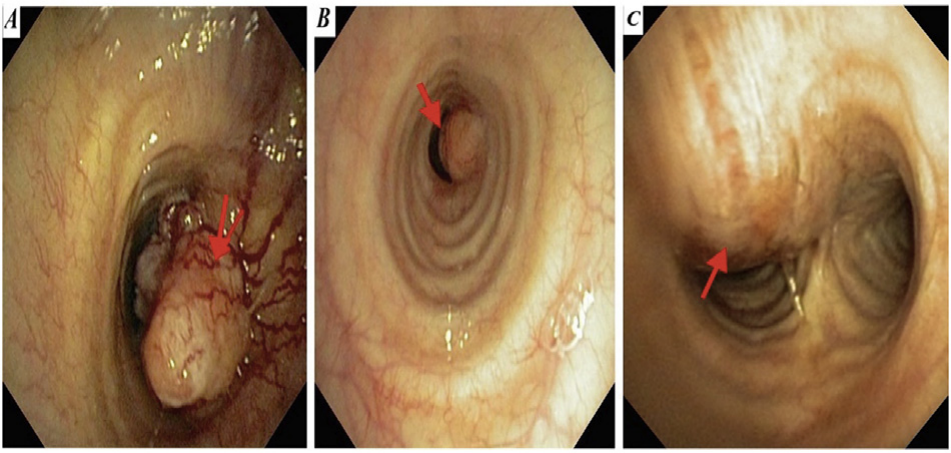
Endoscopic features of tracheal tumors (A: left laterotracheal tumor presenting as a smooth exophytic mass causing tracheal obstruction of 80%. B: Exophytic left tracheal wall mass with almost complete obstruction. C: Tracheal submucosal tumor.

We have 6 men and 2 women in our series. The mean age at diagnosis was 44.4 years range from 17 to 65 years. During the history taking we have found a chronic smoking history in **4** patients, occasional alcohol taking in 2 patients. One patient was first operated with aspergillised cavity of the left lower lobe with hemoptysis confirmed by fibroscopy, with incidental finding of a tracheal tumor that was asymptomatic. Another patient had been treated for asthma considered severe, put under Beta 2imetics and corticosteroid without improvement, the diagnosis was rectified 8 months later, with a finding of a tracheal tumor. One patient had been operated 7 years ago for cervical goiter. The most constant functional sign was dyspnoea found in 7 cases. Two patients had grade IV dyspnea at both inspiratory and expiratory, which had been active for 9 months in the first and 6 months in the later. In another patient, dyspnea was grade III, predominantly inspiratory, evolving for 6 months. Finally, inspiratory dyspnea grade II was noted in 3 cases with respectively duration of evolution 2 months in 2 patients and 6 months in the 3rd patient. Chronic and persistent cough was found in 3 patients, associated with hemoptoid sputum in 2 cases. In 3 cases, wheezing was present, and 2 patients had average abundance haemoptysis. One patient was admitted with bronchorrhea on destroyed lung that had been evolving for several months. The standard chest X-ray showed no tracheal abnormality in all patients. Thoracic computed tomography (CT scan)done in first intention (before fibroscopy) had demonstrated not only the lesion process, but also studied the characteristics of the tumor, especially its distances from the vocal cords and the carina (Fig. 1, Fig. 2), (Table 1). In this series, 7 patients underwent fibroscopy for diagnostic purposes (Fig. 3 A, B and C). Two patients had endoscopic biopsy, one in favour of squamous cell carcinoma and the other was not concluded. Diagnostic fibroscopy was not performed in one patient for safety concerns because he was very uncomfortable with respiratory tract, with a pedicled tumor of the trachea at 5mm from the carena radiologically. In 7 patients, the surgery was performed without histological diagnosis based only on thoracic computed tomography and fibroscopy findings.

**Table 1:** The summary of clinical features of our patients (M = Male, F= Female).

Patients	Age/Sex	Medical history	Clinical signs	CT scan	Fibroscopy	Surgical approach	Surgical procedure	Postoperative course	Histology	Follow-up
1	48/M	Smoking	Hemoptysis	Precarinal right tumor in contact with azygos vein arch	Inflammatory and hemorrhagic round submucosal tumor, biopsy not done	Right posterolateral thoracotomy	Lateral resection and plastic reconstruction in V with anastomosis protection by a pleural graft	Uneventful	Low grade mucoepidermic carcinoma	Uneventful after 72 months
2	17/M		Stage IV dyspnea Expectoration Wheezing Polypnea	Tracheal posterolateral wall tumor (D2-D3) 4 cm from the carina	Smooth and yellowish exophytic tumor crossed by small blood vessels Biopsy not done	Cervicotomy	Tracheal resection and end-to-end anastomosis with right laterotracheal and left paratracheal lymphadenectomy + anastomosis protection by closing of thyroid gland and thymus	Unilateral recurrent laryngeal nerve paralysis.	Adenoid cystic carcinoma	Dead of tracheal radiation stenosis after 8 monts
3	46/M	Smoking Alcohol drinking	Chronic cough Hemoptysis Dyspnea, stage II	4 cm tracheal upper third tumor, 4 cm from vocal cords	Exophytic tumor, bleeding on contact Biopsy not conclusive	Cervicotomy + manubriotomy	Tracheal resection with termino-terminal anastomosis + anastomosis protection by closing of thymic and thyroid gland lobes	Uneventful	Adenoid cystic carcinoma	Uneventful after 42 months
4	44/M	Smoking Severe asthma	Stage III dyspnea Wheezing	Tracheal lower third tumor, 1 cm from the carina	Exophytic, ulcerated mass Biopsy: squamous cell carcinoma	Total sternotomy	Mattey reconstructive surgical technique + mediastinal lymphadenectomy and anastomosis protection by anterior thymic interposition	Unilateral recurrent laryngeal nerve paralysis.	Squamous cell carcinoma	Lymph node metastasis after 60 months, good evolution at present
5	45/F		Expectorations Stage II dyspnea Polypnea Snoring	Right main bronchus polypoid tumor with mediastinal laterotracheal and subcarinal lymphadenopathy	Well vascularised smooth sessile tumor bud Biopsy not done	Right posterolateral thoracotomy	Right pneumectomy and reconstructive tracheobronchial surgery (KERGIN technique) + mediastinal radical lymphadenectomy + anastomosis protection by the thymic lobe	Uneventful	Invasive moderately differentiated adenocarcinoma	Uneventful after 12 months
6	62/M	Smoking History of surgical treatment for lung lower lobe aspergilloma	Chronic cough Expectorations Stage II dyspnea	Tracheal tumor (between the upper and the middle thirds) measuring 3 cm	Huge exophytic bleeding tumor Biopsy not done	Wide cervicotomy + manubriotomy	Tracheal resection with termino-terminal anastomosis + anastomosis protection by closing of thymic and thyroid gland lobes	After 4 days, patient dead of hemothorax due to broncho-arterial fistula	Atypical carcinoid tumor	Dead after 4 days postoperativelly
7	49/M		Stage IV dyspnea Chronic cough Wheezing Snoring	Pedunculated tumor, 5 mm from carina		Right posterolateral thoracotomy	Tracheal resection with termino-terminal anastomosis + anastomosis protection by a thymic graft + mediastinal lymphadenectomy	Uneventful	Pleomorphic adenoma	Uneventful after 3 months
8	30/F	Thyroïdectomy 7 years ago	Dyspnea Wheezing	Endoluminal nodule in the tracheal posterior wall	Subglottic tumor Biopsy not done	Cervicotomy	Tracheal and 3rd cricoid arch resection + thyro-tracheal anastomosis	Uneventful	Ectopic endo tracheal thyroid adenoma	Uneventful at present 7 years

### 3.2 Preoperative management

The in-hospital average length of stay before surgery was 3 days ranged from 1 day to 1 week. During this period, all patients had received antibiotic therapy, respiratory physical therapy with nebulization and humidification of the ambient air, in order to perform a clean and non-inflammatory tracheal surgery.

### 3.3 Anesthetic management

**Fig. 4:**
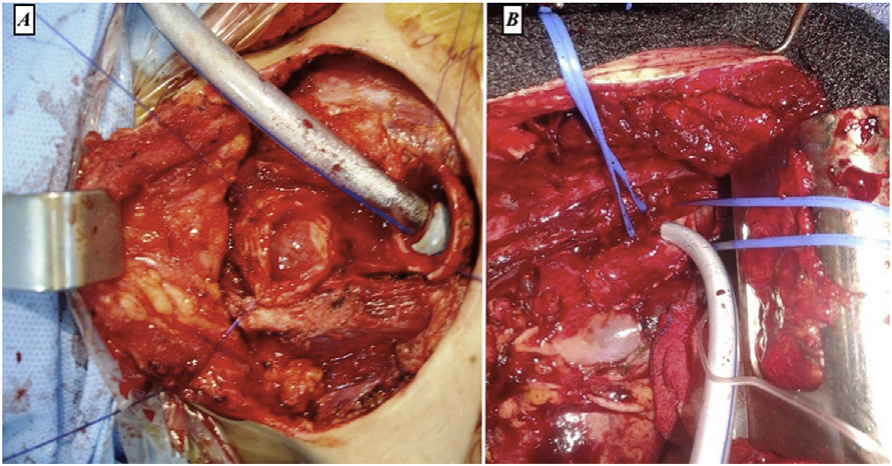
Some intubation procedures (A: surgical perioperative view showing lower tracheal part intubation through the operating field. B: surgical perioperative view showing right main bronchus intubation through the operating field.

All patients had standard operating room monitoring: ECG, non-invasive blood pressure, SPO2, capnography and gastric tube and urinary catheter insertion. The initial anesthesia was short-acting, based on narco-neuroleptanalgesia allowing easy control at the level of anesthesia and usually fast awakening, and having time to intervene in case of desaturation during of intubation. After placement of the intubation probe and inflation of the balloon, the patient is put under deep anesthesia to allow surgery. Ventilation support after tracheal incision was provided by intubation through the operative field. Prior to incision, the orotracheal tube is removed a few millimeters above the tumor. After incision, we performed catheterization of the lower segment of the trachea in 4 patients, and of the left bronchus in 4 patients (Fig. 4A and B). After performing the tracheal anastomosis, the balloon is re-inflated and the tightness of the suture is checked.

**Fig. 5:**
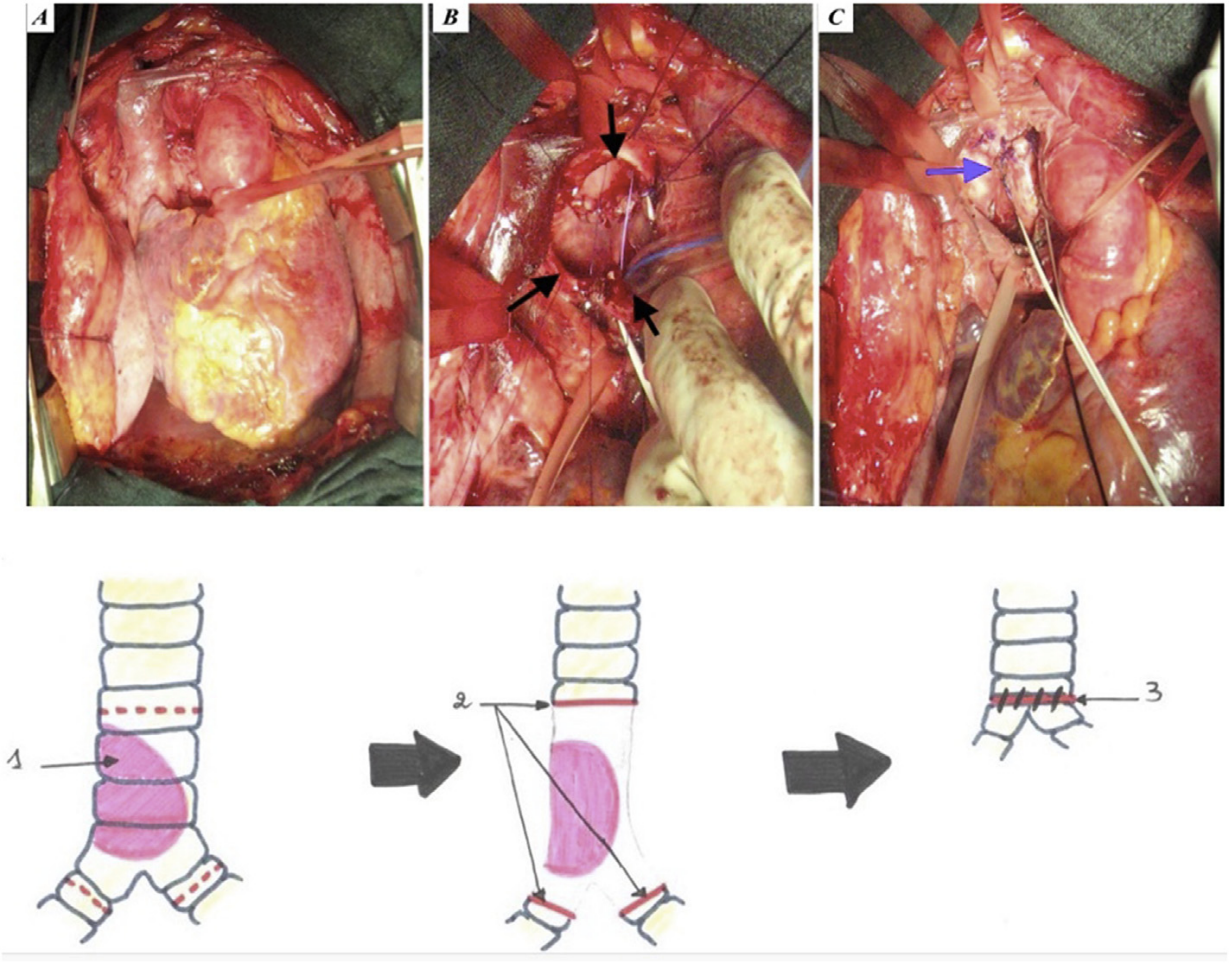
Stages of double barrel type tracheobronchial anastomosis (A: carina exposition through supracardiac vessels. B: anastomosis between the trachea and the 2 main bronchi after the tumor resection with the endotracheal tube in place. C: the end of the procedure showing carina reconstruction by Mattey technique).

**Fig. 6:**
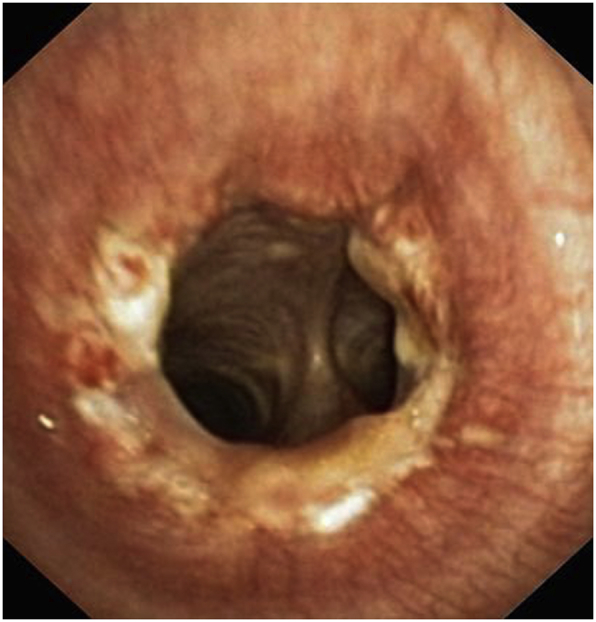
Postoperative endoscopic view showing the healing of the tracheal anastomosis.

### 3.4 Surgical technique

**Diagram 1:**
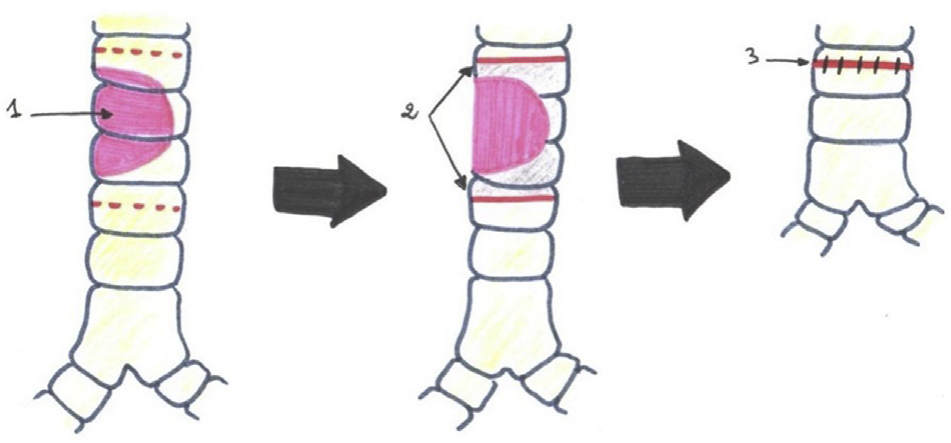
The technique of tracheal resection with end-to-end anastomosis.

**Diagram 2:**
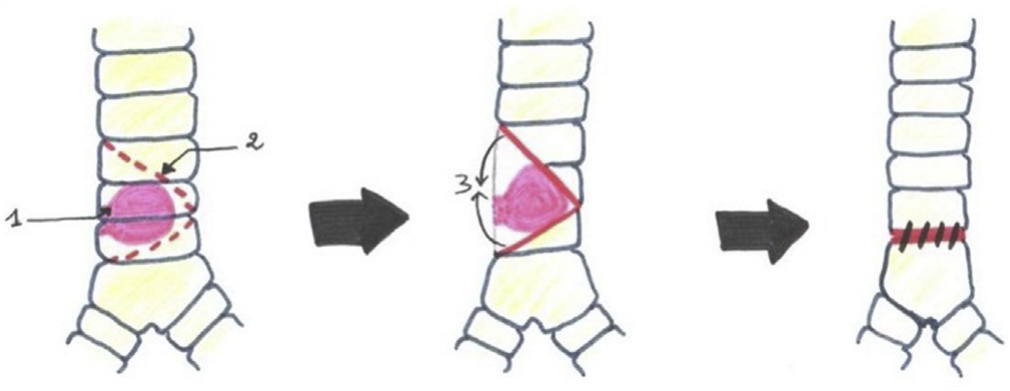
The technique of tracheal lateral resection with plastic reconstruction in ‘‘V’‘.

**Diagram 3:**
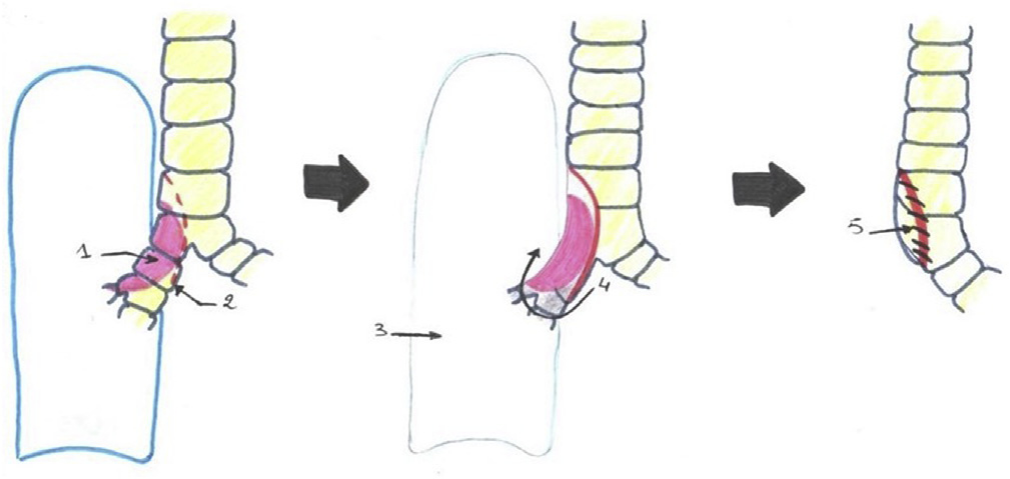
Kergin technique with pneumectomy associated with tracheal plasty reconstruction.

**Diagram 4:**
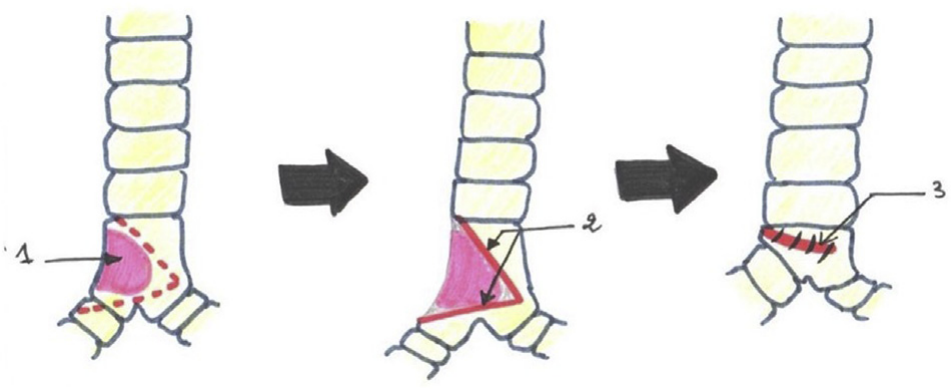
Carinal resection with plasty reconstruction in ‘‘V’.

**Diagram 5:**
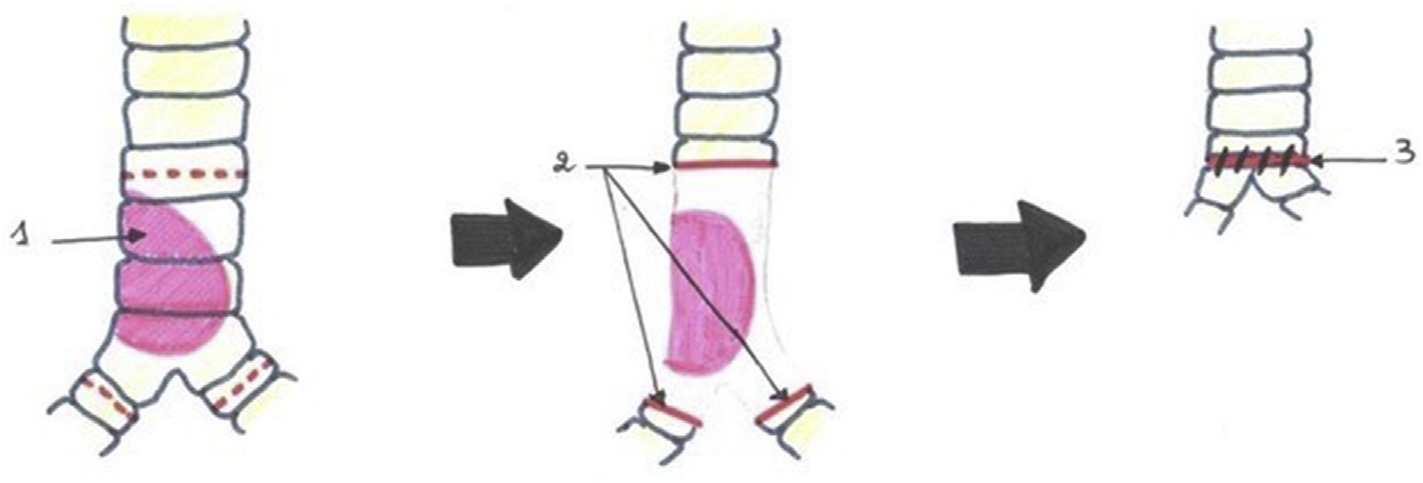
Carina reconstruction by Mattey technique.

In one patient, a large tracheal resection to the anterior arch of the cricoid cartilage with thyro-tracheal anastomosis has perfomed. Three of our patients had undergone end-to-end tracheal resection-anastomosis (Diagram 1). In 3 other patients, a tracheal plasty was performed: a lateral resection with “V” plasty in 1 case (Diagram 2), a KERGIN type bronchial plasty after right pneumonectomy in 1 case (Diagram 3).), and a resection on the hull with “V” plasty (Diagram 4) was performed in the third patient. One patient had undergone tracheal resection enlarged to the hull with tracheobronchial anastomosis ‘‘in double barrel of rifle’’ by MATTEY technique (Fig. 5, Diagram 5). The resected trachea average length was 4.5cm ranged from 4cm to 5cm. The mobilization technique used in our patients was dissection and release of the trachea. We have protected the tracheal anastomosis with surrounding tissues namely the thyroid and the thymus in 4 patients, the thymus in 2 patients and a pleural flap in 1 patient. The ganglionic dissection had been in 5 patients. In all patients, provisional sterno-chin fixation was performed to keep the neck in flexion, in order to avoid disruption of the tracheal anastomosis.

### 3.5 Pathological findings

The most common histological type in our series was Adenoid Cystic Carcinoma in 2 of our patients. For the other patients we have found squamous cell carcinoma (1 case), adenocarcinoma (1 case), atypical carcinoid tumor (1 case), low grade mucoepidermoid carcinoma (1 case), an adenoma pleomorph (1 case) and endotracheal goiter (1 case). All ganglionic dissection was negative.

### 3.6 Operative suites

All patients were extubated on the operating table with prolonged monitoring in the recovery room, before transferring them systematically in the intensive care unit. The follow-up protocol in the intensive care unit consisted of an upright position with flexion of the head forward, and monitoring of clinical constants, post-operative respiratory physiotherapy with Non invasive ventilation sessions, humidification of the air, oxygen therapy, incentive spirometry and mouth aspirations. Prophylactic antibiotic therapy, gastric protection and prevention of thromboembolic disease have used in all patients. Oral feeding was resumed from the 2nd-3rd day. In the immediate postoperative period, we registered 2 cases of recurrent paralysis and 1 case of death occurring on fourth postoperative day following a cataclysmic haemothorax. Endoscopic control systematically on tenth post-operative day was satisfactory. A second endoscopic control was performed on 30^th^ postoperative day and no case of stenosis or granuloma was noted (Fig. 6).

### 3.7 Adjuvant therapy

Only two patients had adjuvant therapy following a decision in a multidisciplinary consultation meeting. He had benefited from postoperative radiotherapy. For the second patient, it was a squamous cell carcinoma that was sanctioned with radiochemotherapy.

### 3.8 Morbi-mortality

We have noted 2 cases of postoperative recurrent palsy following mediastinal lymph node dissection. Their management consisted of speech therapy, complete recovery of phonation, and normal swallowing. The postoperative course was simple for the other patients. We have registered 1 case postoperative death. This was the patient who was initially operated on aspergillated cavity of the left lower lobe by lobectomy, and reoperated on 20th postoperative day for the tracheal tumor discovered by chance, and died on 4th day of tracheal surgery in a table of cataclysmic hemothorax following Broncho-arterial fistula complicating left lower lobectomy.

### 3.9 Long term evolution

A patient undergone surgery for squamous cell carcinoma of the trachea invading the hull, who is still alive, presented a cervical ganglionic relapse after 5 years, he has undergone cervical lymph node dissection by cervicotomy, followed cervical radiotherapy. One year later, the chest CT scan showed 2 newly emerging pulmonary micronodules requiring post-discussion monitoring decided after a multidisciplinary consultation meeting. Another patient operated for adenoid cystic carcinoma with invasion of the lower resection margin had post-radiation tracheal stenosis. He had repeatedly undergone endoscopic treatment with tracheal prosthesis in order to dilate the stenosis, the last one was complicated by tracheal perforation which led to death in a respiratory distress month later the initial surgery. After an average follow-up of 32 months in the 6 patients who are alive year to date and regularly seen in consultation, no complication has been noted so far.

## 4 Discussion

In adult, about 90% of primary tracheal tumors are malignant while in children only 10%–30% are malignant. The incidence of malignant tracheal tumors is approximately 0.1 per 100,000 populations per year, corresponding to approximately 0.2% of all tumors of the respiratory tract, and 0.02%–0.04% of all tumors in all localizations. Malignant tumors of the larynx and bronchi are approximately 40 and 400 times respectively more frequent than tracheal cancers [6,7]. Squamous cell carcinoma and cystic adenoid carcinoma represent respectively 35% and 40% of adult tracheal cancers, respectively [8,9]. In the context of adenocarcinoma, smoking is certainly associated in genesis of precancerous lesions [10]. In our study, we found no correlation between smoking and the histological types found. Adenoid cystic carcinoma generally affects patients in their forties, these patients are younger than those affected by squamous cell carcinoma [11]. Carcinoid tumors are more common in the fourth decade of life [11]. In our study, the average age at diagnosis was 44 years and 31.5 years for adenoid cystic carcinoma. However, we noted 1 case of adenoid cystic carcinoma in a 17-year-old patient. The peculiarity of tracheal tumors is the latency of clinical symptoms. They have usually a slow and progressive growth, with nonspecific symptomatology in general such as dyspnea, wheezing or an insidious cough installation. The clinical feature can lead to asthma or COPD misdiagnosis, wrongly treated with B2 mimetics and corticosteroids, with a normal chest X-ray in most cases. In the series of Brayan et al. 20% of tracheal tumors were treated as late-onset asthma [12]. The delay in the diagnosis is due to the tumor extension at the time of the care. The tumor usually grows until it reaches 50% of the size of the trachea caliber before dyspnea is evident. The combination with hemoptysis suggest squamous cell carcinoma, but can also be seen in all other types of tumors. In our series, a patient with moderate to severe dyspnea was wrongly treated for asthma considered severe and put under inhaled B2 mimetics and corticosteroids without improvement. Thus any severe asthma in the adult who does not respond to the usual treatment should evoke a tumor of the trachea and start a management approach. Thoracic CT is a diagnostic step that can be performed before endoscopic explorations. It assesses the localization, the size, the extent of the lesion in relation to the vocal cords and the hull, its circumference, the infiltration of the mediastinum, but also the search for local or regional metastases while appreciating the quality of pulmonary parenchyma [13]. It also makes it possible to evaluate the degree of tracheal obstruction and to assess the utility of endoscopy, which is usually rigid, for diagnosis (biopsies) or therapeutic purposes (unclogging). However, conventional radiology techniques may underestimate the parietal extension of the tumor. In the prospective series reported by Shadmehr et al., 9% of tumors found to be CT resectable were finally found to be unresectable for surgical exploration [14]. Latest advances in multibrand CT techniques have allowed three-dimensional reconstructions and virtual tracheobronchoscopies to identify intra- and extraluminal components, pedicle lesions sessile over several centimeters [15]. Three-dimensional reconstruction techniques offer a useful alternative to bronchoscopy as a screening tool for post-surgical recurrence [13]. The use of new aerosolized contrast agents allows differentiation between benign and malignant mucosal lesions [16]. Endoscopy is a crucial step in the management of tracheal tumors. In fact there are 2 types of tracheobronchoscopy; bronchoscopy flexible and rigid and each of these 2 techniques has an indication and a particular use. The rigid bronchoscopy is essential in the diagnostic and therapeutic management of the tracheal tumors making it possible to evoke that they are benign or malignant in case of abnormal aspect of the mucous membrane. This technique can allow to treat complications such as bleeding, fistulas or obstruction of the tracheal lumen. It can also allow to place an endotracheal prosthesis in palliative management [17]. However, it should be noted that fibroscopy does not always provide the histological diagnosis because the biopsy is not always easily feasible. However, fibroscopy does not always provide the histological diagnosis because the biopsy is not always easily feasible. The histological diagnosis will be determined on the surgical excision specimen. Literature have shown that interventional endoscopy aimed at palliative was of no interest in patients seeking surgical treatment [18]. Thanks to the progress made in recent years, the majority of tracheal pathologies are now treated by direct anastomosis resection. This chirurgical technique is usually performed by cervicotomy, total or partial cervico-sternotomy, rarely by right posterolateral thoracotomy [19]. The dissection technique consists of a delicate release of the area to be resected while preserving the micro vascularization of the 2 segments that are candidates for anastomosis [20]. In the same way as surgery, anesthetic techniques have also evolved with the introduction of intubation in the operative field, “jet ventilation” or extracorporeal circulation including ECMO “Extra Corporeal Membrane Oxygenation” [21]. The termino-terminal anastomosis often requires a plasty in case of a problem of congruence of caliber. Several artifices of tracheal mobilization (intraoperative cervical flexion, high cervical mobilization or low thoracic mobilization) make it possible to minimize the effect of the tension at the level of the anastomosis area. In the end, tumor localization at certain sites such as the hull requires a technique of specific reconstructions, which varies according to the schools [22]. Surgery is contraindicated when the complete resection of the tumor must exceed 50% of the length of the trachea, in order to avoid excessive anastomotic tension [19]. In the case of cystic adenoid carcinoma, which has a very slow development, a surgical resection guided by radiotherapy can be considered with a definite benefit for the patient [23]. Indeed, radiotherapy can be used in the case of tracheal tumors either adjuvant therapy after surgical resection, or in the first line in case of unresectable tumor. In adjuvant therapy, radiotherapy improves survival by reducing the risk of local relapse [24,25]. Radiotherapy in adenoid cystic carcinoma is the best documented in the literature [26]. The 5-year survival rate varies in the surgical series, with or without radiotherapy, between 65 and 85% [[27], [28], [29], [30]]. Given the possibility of local recurrence and/or the occurrence of late metastatic disease, some authors systematically advocate postoperative radiotherapy, while others only propose it when the margins of resection are invaded [26,27,30]. In general, tracheal tumors have a poor prognosis, with 57% survival after surgical treatment, and 6–11% in patients treated with radiotherapy alone and all histological types combined [7]. Thus, the prognostic factors identified are early diagnosis, tumor stage, histological type and therapeutic choice [6,[31], [32], [33]]. Selection of patients for definitive surgery is the most important factor in improving the prognosis for patients with primary tracheal malignancy [6,7,34].

## 5 Conclusion

Tracheal tumors have a low clinical course with non specific symptoms leading to late diagnosis. The clinical management of patients with these tumors is challenging as it requires specific and accurate surgical techniques. Nowadays, rigid bronchoscopy is a valuable therapeutic tool in the management of tracheal tumors. Surgical procedures with tracheal resection and anastomosis remains a better therapeutic option with good prognostic outcomes.

## Ethical approval

There is no ethical committee in our country (Not applicable for this manuscript).

## Sources of funding

There are no sources of funding.

## Authors’ contributions

All authors contributed to the study design. SR, HH and ML performed data collection and data analysis. SR, EB and HH performed the cost analysis. All authors critically interpreted all data analysis. YO and MS composed the manuscript, and all the remaining authors provided critical edits to the final draft. All authors read and approved the final manuscript.

## Conflicts of interest

There are no conflicts of interest.

## Research registration number

Researchregistry4721.

## Guarantor

Sani Rabiou.

## Availability of data and materials

The datasets used and/or analysed during the current study available from the corresponding author on reasonable request.

## Consent for publication

Not applicable.
